# Antimicrobial Resistance Profiles of *Escherichia coli* Isolated from Broiler and Layer Chickens in Arusha and Mwanza, Tanzania

**DOI:** 10.1155/2021/6759046

**Published:** 2021-10-22

**Authors:** Ruth W. Kiiti, Erick V. Komba, Peter L. Msoffe, Stephen E. Mshana, Mark Rweyemamu, Mecky I. N. Matee

**Affiliations:** ^1^Department of Veterinary Medicine and Public Health, Sokoine University of Agriculture, P.O. Box 3021, Morogoro, Tanzania; ^2^Catholic University of Health and Allied Sciences, P.O. Box 1464, Mwanza 33109, Tanzania; ^3^SACIDS Africa Centre of Excellence for Infectious Diseases, Sokoine University of Agriculture, P.O. Box 3297, Morogoro 67125, Tanzania; ^4^Muhimbili University of Health and Allied Sciences, P.O. Box 65001, Dar es Salaam 11103, Tanzania

## Abstract

The rise in the spread of antibiotic-resistant pathogens such as *Escherichia coli* is one of the very important dynamics off-putting treatment and prophylaxis possibilities, hence posing a threat to the modern human medicine, veterinary medicine, and food safety. Therefore, the aim of this study was to determine antimicrobial resistance profiles in *E. coli* isolates obtained from broiler and layer chickens in Mwanza and Arusha regions in Tanzania. A cross-sectional study was carried out from February to March, 2021, in 402 poultry farms in Mwanza (201) and Arusha (201) regions in Tanzania. All samples that tested positive for *E. coli* were confirmed using MALDI-TOF MS, and two hundred and four (204) *E. coli* isolates were randomly chosen and subjected to antimicrobial susceptibility testing by disc diffusion method. Data were entered in Microsoft Excel^®^ and analyzed using SPSS version 20. Isolates were tested against seven antimicrobial agents belonging to seven classes of antimicrobials. All the tested isolates (*n* = 204) were resistant to at least one antimicrobial agent. Overall, the highest resistance was observed in ampicillin (100%), whereas the lowest resistance was recorded for gentamicin (10.3%). Majority of the isolates (86.76%) were multidrug resistant. Antimicrobial resistance of *E. coli* to four classes of antimicrobial agents was the highest in this study (31.1%). Six of the 177 tested isolates (2.9%) were resistant to the seven classes of antimicrobial agents. 21 of the 204 (10.29%) isolates were ESBL producers where 21/21 (100%) isolates expressed *bla*_TEM_ genes and only two isolates expressed (2/21) *bla*_CTX-M_ gene. The isolates obtained in this study displayed high resistance to commonly used antimicrobial agents in veterinary and human medicine. This implies that there is existence of practices that accelerate antimicrobial resistance in the production of the sampled birds and therefore integration of appropriate use of antimicrobial agents and other measures that curb the spread of resistant genes is necessary.

## 1. Introduction

Antimicrobial resistance (AMR) is a state whereby microbial pathogens develop resistance mechanisms toward evading antimicrobial drugs [1, 2]. The process is accelerated by the influx of antimicrobial resistance genes (ARGs) to the environment from livestock and human wastes and by the vast quantities of antibiotic residues discharged from the pharmaceutical industry, hospitals, and intensive livestock farms [3–5]. It has been approximated that 4.2 million deaths do occur annually in Africa due to antimicrobial resistance, and 300 million people are expected to die prematurely worldwide due to drug resistance over the next 35 years [3]. The gross domestic product (GDP) of the whole world will be lowered by 2 to 3.5% by 2050, which is translated into losing 60 to 100 trillion USD if the antimicrobial resistance issue will not be tackled [3]. Antimicrobial drugs have increasingly been applied or administered as preventive chemotherapy [6, 7] and growth promoters [8] in livestock production industries such as dairy farms, poultry farms, and pig farms as well as many other industries globally. The worldwide regulatory authorities, that are, European Union, 2010, and Codex Alimentarius Commission, 2012, highlighted guidelines on veterinary drug usage, including drug withdrawal period and maximum residue level, in order to assure consumers on the safety of the livestock products. Globally, the antimicrobials use in animals is estimated to increase by 67% (from 63, 151 to 105, 596 tonnes) between 2010 and 2030 as a result of increased use of antimicrobial agents on foods of animal origin and to more intensive animal farming practices in middle-income countries [9].

*Escherichia coli* is a commensal bacterium, but some of its strains have developed a potential to cause intestinal and extra-intestinal infections [10] and have also developed resistance to some antimicrobial agents [11]. These disease-causing and antimicrobial-resistant strains of *Escherichia coli* pose a threat to public health and animal health as well as food safety if they develop antimicrobial resistance mechanisms [2].

AMR remains a growing problem in humans, animals, and the environment in Tanzania [12–14]. Tanzania has three major poultry production systems: traditional indigenous, improved family chicken, and commercial specialized chicken systems [15]. The population of chickens in the country is estimated at 72 million, of which 40 million are indigenous chickens and the remaining 32 million are exotic poultry, which includes over 24 million broilers and 8 million layers [16]. Apparently, the consumption of poultry meat, more so broiler chicken meat, is far higher than other meat types in the country. At the end of their production cycle, layer chickens also enter the food chain as spent birds. This has implications on possibilities of wide spread human health effects in case these chickens are colonized with zoonotic pathogens especially when the pathogens are AMR. Through limited studies [17–19], antimicrobial resistance has been detected among isolates from chicken in the country. Because of the shared risk of AMR between animals and humans, One Health approach [20] is critically needed to enhance the integration of information about the resistance of microorganisms circulating in humans, animals, and the environment to inform appropriate actions [21].

This study aimed at investigating the occurrence and antimicrobial resistance profiles of *Escherichia coli* isolates from broiler and layer chickens in Arusha and Mwanza, Tanzania. It is anticipated that this study will bring to the fore the spread of antimicrobial resistance in *E. coli* from broilers and layers to humans. Besides documenting the resistance, data generated from this study supplement the existing antimicrobial resistance profiles of *E. coli* isolates from broilers and layers in Tanzania.

## 2. Materials and Methods

### 2.1. Study Area

**Figure 1:**
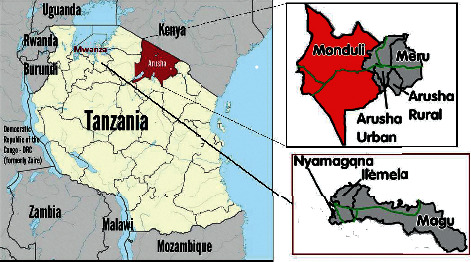
A map showing the study area.

The study was carried out in Arusha and Mwanza regions. The regions are known to be high chicken-producing (broilers and layers) areas in Tanzania with deviations in production existing between them. In these regions, the demand and consumption of poultry meat and poultry products are high, with a human population of 1,694,310 and 2,772,509, respectively [22]. The selected districts in Mwanza were Ilemela, Nyamagana, and Magu; whereas in Arusha, the districts were Arusha Urban, Arusha Rural, Meru, and Monduli (Figure 1).

### 2.2. Study Population

#### 2.2.1. Inclusion Criteria

Chickens that were about to enter the food chain were sampled in this study. They included broilers that reached the slaughter weight and culled layer hens that had reached the end of their laying period and were being considered to be sold for human consumption (spent layers). The focus was on commercial and semicommercial broiler and layer production systems to capture resistance profiles in these different systems.

#### 2.2.2. Exclusion Criteria

Sick chickens (broilers and layers) were excluded from sampling, as they could not represent the status of resistance in bacteria carried by chickens that enter the food chain. Broilers and layers that had been treated within the past seven days were also excluded from the study.

### 2.3. Selection of Sampling Farms

Farms were selected based on having more than 100 broilers and/or layer chickens kept for commercial purpose. A distance of 100 meters to 150 meters from one farm to another was considered in this study.

### 2.4. Sampling Design

Sampling was conducted mainly directly from farms. Information was obtained regarding the farm, and the sampling frame was composed of broiler and layer chicken farms; the sampling units were broiler and layer chickens.

### 2.5. Sample Size

The sample size was determined using the single proportion formula:  (*n*)=*Z*^2^*Pq*/*d*^2^, where (*n*) is the required sample size, *Z* = *Z* value for a given confidence level, *p* = expected prevalence, *q*=(1 − *p*) and *d* = allowable error of estimation. The confidence level was assumed to be 95% with an allowable error of 5%, and thus, *Z* was 1.96. Prevalence of 50% was used in the calculation, which resulted in *n* = 384 as the minimum sample size of the chicken.

### 2.6. Isolation and Identification of *Escherichia coli*

The cloaca swab sample was placed in the maximum recovery diluent transport media, shaken well, and transported to the laboratory. A loop full of the cloaca swab sample was obtained from the maximum recovery diluent media, and streaks were made on MacConkey agar (Oxoid Ltd., Basingstoke, UK) and incubated aerobically at 37°C for 18–24 hours. The presumptive colonies obtained were purified by subculturing on blood agar (Oxoid Ltd., Basingstoke, UK) at 37°C for 18–24 hours aerobically. The pure colonies were then subjected to gram staining and biochemical tests for indole methyl red, Voges–Proskauer, and citrate utilization (IMViC; Sigma-Aldrich Co., Switzerland) according to the manufacturer's instructions to identify *E. coli*. The identified pure isolates were preserved. Isolates that indicated indole positive, methyl-red positive, Voges–Proskauer negative, and citrate negative were established as *E. coli*, then preserved in Mueller Hinton Broth containing 15% v/v glycerol, and stored at −20°C for antimicrobial susceptibility testing. The obtained *E. coli* isolates were further confirmed using matrix-assisted laser desorption-ionization time-of-flight mass spectrometry (MALDI-TOF/MS) [23].

### 2.7. Antimicrobial Susceptibility Testing

Antimicrobial susceptibility testing of bacterial isolates was performed in accordance to the guidelines highlighted by the Clinical and Laboratory Standards Institute (CLSI, 2018) adopting the disc diffusion method on Muller Hinton (MH) agar (Oxoid Ltd, Basingstoke, UK), as described by Luangtongkum et al. [24]. The preserved pure *E. coli* isolates were recuperated on MacConkey agar, purified in blood agar and nutrient agar (Oxoid Ltd., Basingstoke, UK), and then subjected to sensitivity tests. A single pure colony was obtained and suspended in 2 ml of sterile normal saline, and its turbidity was adjusted to that of the standard 0.5 McFarland (Oxoid Ltd., Basingstoke, UK). Thereafter, a sterile swab was dipped in the suspension and used to inoculate the *E. coli* suspension on Mueller Hinton agar (Oxoid Ltd., Basingstoke, UK). Afterward, the antibiotic discs were placed on the inoculated Mueller Hinton agar plate using a q1BBL Sensi-Disc dispenser (Fisher Scientific, UK) and then incubated at 37°C for 18–24 hours. The zones of inhibition were measured in millimeters using a ruler; then, the results were interpreted using the CLSI guidelines 2018 and also the manufacturer's instructions. The isolates were documented as susceptible (S), intermediate (I), or resistant (R) according to clinical breakpoints defined by CLSI together with the zone sizes (mm). The most commonly used antimicrobial agents in livestock and humans in the study area and others recommended by WHO [2] were tested for resistance; they included, carbapenems (ertapenem 10 *μ*g), phenicols (chloramphenicol 30 *μ*g), cephems (ceftriaxone 30 *μ*g), quinoles and fluoroquinoles (ciprofloxacin 5 *μ*g), penicillin (ampicillin 10 *μ*g), folates (trimethoprim/sulfamethoxazole 25 *μ*g) and aminoglycosides (gentamicin 120 *μ*g). *E. coli* ATCC 25922 was used as a reference strain and for quality control purposes. Proportions of isolates that were resistant to any number of antimicrobial agents were reported. An isolate that was resistant to three or more classes of antimicrobial agents was referred to as multidrug resistant as defined by Magiorakos et al. [25].

### 2.8. Screening of Extended-Spectrum Beta-Lactamase-Producing *E. coli*

The extended-spectrum beta-lactamase-producing *E. coli* were determined phenotypically using cefotaxime (CTX-30 *μ*g) alone and cefotaxime/clavulanic acid (CTX/CLA-30 *μ*g/10 *μ*g) and ceftazidime (30 *μ*g) alone and ceftazidime/clavulanic acid (10 *μ*g) as recommended by CLSI 2018 using the disk diffusion method. A difference in distance of ≥5 mm increase in the zone of diameter was termed as indicating ESBL-producing *E. coli*.

### 2.9. DNA Extraction

The DNA was extracted by boiling method at 100°C for 10 minutes. The Eppendorf tubes containing the mixture were suspended in boiling water for 10 minutes; then, they were removed and cooled in a freezer for 5 minutes, then reboiled for 10 minutes, and afterward cooled for 5 minutes in a freezer. This was followed by centrifugation of 1500 rpm for 3 minutes.

### 2.10. Polymerase Chain Reaction Analysis for bla_SHV_ and bla_CTX-M_

Master mix was prepared for all the phenotypic ESBL-positive samples by mixing PCR premix (12.5 *μ*L), forward primer for *bla*_SHV_ (F-ATG CGT TAT ATT CGC CTG TG) and *bla*_CTX-M_ (0.5 *μ*L each) (F-5′-SCS ATG TGC AGY ACC AGT AA), reverse primer for *bla*_SHV_ (R-AGC GTT GCC AGT GCT CGA TC) and *bla*_CTX-M_ (0.5 *μ*L) (R-5′-CCG CRA TAT GRT TGG TGG TG), and then 2.5 *μ*L of nuclease-free water was prepared and brought up to the volume of 21 ESBL samples. Then, it was aliquoted to the PCR tubes, and thereafter 3 *μ*L of each extracted DNA was added to the PCR tubes containing the master mix. ESBL *bla*_CTX-M_ and *bla*_SHV_ were identified using multiplex PCR with the following PCR amplification conditions: initial denaturation at 95°C for 5 minutes, then denatured for 30 cycles at 94°C for 30 seconds, annealing was done at 58°C for 30 seconds, an extension was performed at 72°C for 2 minutes, and then the final extension was set at 72°C for 10 minutes [26–28].

### 2.11. Molecular Identification of bla_TEM_

Master mix was prepared for all the phenotypic ESBL-positive samples by mixing PCR premix (12.5 *μ*L), forward primer (F-ATG AGT ATT CAA CAT TTC CG) for *bla*_TEM_ (0.5 *μ*L each), reverse primer (R-CCA ATG CTT AAT CAG TGA GG) for *bla*_TEM_ (0.5 *μ*L), and then 3.5 *μ*L of nuclease-free water was prepared and brought up to the volume of 21 phenotypically ESBL-positive samples. Then, it was aliquoted to the PCR tubes, and thereafter 3 *μ*L of each extracted DNA was added to the PCR tubes containing the master mix. ESBL *bla*_TEM_ genes were identified using uniplex PCR with the following PCR amplification conditions: initial denaturation at 95°C for 5 minutes, then denatured for 30cycles at 94°C for 15 seconds, annealing was done at 58°C for 30 seconds, an extension was performed at 72°C for 2 minutes, and then the final extension was set at 72°C for 10 minutes [26].

### 2.12. Gel Electrophoresis Preparation

Working buffer was prepared by measuring 980 ml of distilled water and was mixed with 20 ml of TAE buffer stock solution in a conical flask. Afterward, 1% agarose gel was prepared by weighing 1 gram of agarose, which was dissolved in 100 ml of the working TAE buffer. The working TAE buffer was poured into the gel tank, wells were made, and the gene bands were visualized using 2.5 *μ*L of ethidium bromide dye, and the gel image was documented using the BIOBASE PCR Gel Document imaging system for DNA. Those that produced bands were termed positive, whereas those that did not show bands were termed as negative.

### 2.13. Data Analysis

Data were captured in Microsoft Excel^®^, and the chi-square test was carried out to give proportions of resistance using SPSS, version 20. Statistical significance was set at *p*-value < 0.05.

## 3. Results

**Figure 2:**
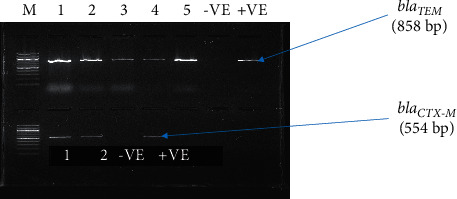
Amplified *bla*_TEM_ and *bla*_CTX-M_ genes of five out of 21 ESBL-positive samples; where samples 1 and 2 expressed both ESBL genes, while samples 3–5 expressed the *bla*_TEM_ gene. *M* = Marker 100 bp DNA ladder, 1–5 = samples, −VE = negative control, +VE = positive control, and bp = base pair.

A total of 402 cloaca swabs were collected from 402 broiler and layer chicken farms in Arusha and Mwanza regions in Tanzania. All samples (100%) tested positive for *Escherichia coli*. Random selection of the isolated *E. coli* was performed, and 204 (50.7%) isolates were chosen and then subjected under antimicrobial sensitivity tests. Out of the selected isolates, 103 (50.5%) were from broilers and 101 (49.5) were from layer chickens. The randomly chosen isolates from the Arusha region were 102 (50%), and 102 (50%) were from the Mwanza region in Tanzania. Twenty-one isolates tested positive for ESBL phenotypically and through molecular characterization, with all 21/21 (100%) isolates expressing *bla*_TEM_ genes and only two isolates (9.5%) expressed *bla*_CTX-M_. No isolate had the *bla*_SHV_ gene in this study (Table 1 and Figure 2). Overall, the highest resistance was observed in ampicillin (100%), whereas the lowest resistance (10.3%) was recorded for gentamicin. Frequencies of resistance against other antimicrobial agents are as presented in Table 2. There was no significant variation of antimicrobial resistance by chicken type (broiler and layer) against the tested antimicrobial agents, as shown in Table 3.

**Figure 3:**
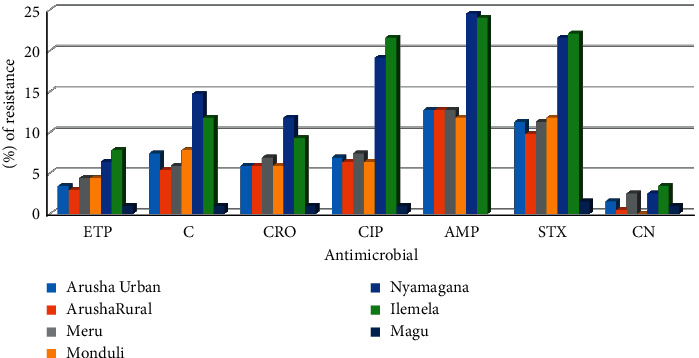
Prevalence of resistance to selected antimicrobial agents among *Escherichia coli* isolates obtained from broiler and layer chickens in Arusha and Mwanza by district (%). ETP = ertapenem, C = chloramphenicol, CRO = ceftriaxone, CIP = ciprofloxacin, AMP = ampicillin, STX = trimethoprim/sulfamethoxazole, CN = gentamicin.

As shown in Table 3, broilers had higher resistance to all selected antimicrobial agents, though the difference was statistically insignificant. The highest resistance to ertapenem drug was recorded in the Ilemela district, and the Nyamagana district recorded the highest resistance to chloramphenicol (14.7%), ceftriaxone (11.8%), and ampicillin (24.5%). The highest resistance to ciprofloxacin (21.6%) and gentamicin (3.4%) was recorded in the Ilemela district. Trimethoprim/sulfamethoxazole drug indicated the lowest prevalence in the Magu district. The antimicrobial resistance of *Escherichia coli* to the selected antimicrobial agents by the district was not statistically significant except for ciprofloxacin (*p*=0.001) (Figure 3).

Twenty-seven out of two hundred and four *E. coli* isolates (27/204, 13.24%) did not exhibit multidrug resistance patterns. Of these, 5.4% showed resistance to only one class of antimicrobial agents, whereas 7.8% depicted resistance to at most two classes of antimicrobial agents (Table 4).

One hundred and seventy-seven (86.76%) selected *E. coli* isolates showed resistance to three or more antimicrobial agents belonging to three or more different classes of antimicrobial agents. Resistance to four classes of antimicrobial agents was the highest with a prevalence of 31.1% followed by resistance to 3 classes of antimicrobial agents (28.2%). Multidrug resistance to 7 classes was the lowest (3.4%), as shown in Table 5.

The most common pattern of multidrug resistance was observed in 28 selected *E. coli* isolates in both Mwanza and Arusha (ETP, C, CRO, CIP, AMP, STX) followed by (C, CIP, AMP, STX), where 20 isolates depicted a similar resistance pattern to four classes of the tested antimicrobial agents, and the third most observed resistance pattern was (CRO, CIP, AMP), as shown in Table 6.

The most prevalent extended-spectrum beta-lactamase gene in this study was *bla*_TEM_, whereas there is no detected *bla*_SHV_ gene as shown in Table 1 and Figure 2.

The most common pattern observed contained a combination of the following antimicrobial agents CRO, AMP, STX. One isolate among the ESBL-positive isolates showed resistance to all the seven antimicrobial agents tested in this study (Table 7).

## 4. Discussion

The overall prevalence of multidrug-resistant *E. coli* isolates according to this study was 86.76% (177/204) in both Mwanza and Arusha, which was significantly higher than that in a study conducted in Dar es Salaam where the MDR prevalence of broilers and layers was 69.3% (147/212) [29] and 51.6% as reported by Kimera et al. [30]. This prevalence of MDR *E. coli* isolates is also higher than the one (63.4%) obtained from local and imported retail chicken carcasses in Qatar [31] and also higher than 68% MDR *E. coli* isolates obtained from healthy chicken farms in the region of Dakar, Senegal [32]. Rahman et al. [33] also reported a slightly lower prevalence of multidrug-resistant *E. coli* isolates obtained from broiler and layer chickens, which was 49.23% for broilers and 51.09% for layer chicken in Sylhet Division, Bangladesh.

The *E. coli* isolates showed differences in resistance against the different antimicrobial agents used in this study. The highest resistance was observed in ampicillin (100%); a similar observation was made by Seo and Lee [34] in Korea and Igizeneza et al. [35] in Nairobi. Higher resistance levels were also recorded for trimethoprim/sulfamethoxazole (89.2%), ciprofloxacin (68.6%), and chloramphenicol (53.9%). The high resistance could be because of the lower prices for these antimicrobial agents and also the availability of the antimicrobial agents in Arusha and Mwanza regions, which make the poultry farmers to easily afford them as suggested by Aworh et al. [36], whose study was based in Nigeria, by Baran et al. [37] in Eastern Turkey, and by Musa et al. in Italy [38]. A similar trend of antimicrobial resistance was observed by Rugumisa et al. [19] in Arusha District where resistance to sulfamethoxazole was the highest (80.3%); though for ampicillin, the prevalence in this study significantly differs from Rugumisa et al. [19] findings (49.1%), which most probably could mean that the overuse and misuse of ampicillin have increased in the Arusha region. A high prevalence of antimicrobial resistance to ampicillin was also observed in *E. coli* isolated from frozen chicken meat in Bangladesh by Parvin et al. [39]. In this study, resistance was observed in a group of antimicrobials known as carbapenems, which include ertapenem and meropenem, among others, which is not in tandem with a study carried out in chicken meat in Eastern Turkey where resistance to carbapenems was not observed [37]. A high susceptibility level of the isolated *E. coli* to gentamicin was observed in this study, which does not augment with a study done in Eastern Turkey by Baran et al. [37] and Aworh et al. [36] in Nigeria. A similar lower resistance to aminoglycosides was also observed in studies carried out in Tunisia by Abbassi et al. [40] and in Korea by Seo and Lee [34]. The prevalence of ESBL-producing *E. coli* isolated from the selected *Escherichia coli* in this study was 10.29% (21/204), with 100% having *bla*_TEM_ gene and 9.52% had *bla*_CTX-M_ gene; no isolate expressed *bla*_SHV_ gene. The prevalence of ESBL-producing *E. coli* in this study was lower than the findings of Chishimba et al. [41] whose research in Zambia recorded a higher prevalence of 20.1%. This prevalence was not in tandem with Kimera et al. [30], where 80% of the ESBL gene was *bla*_CTX-M_ with no observed *bla*_TEM_ and *bla*_SHV_ ESBL genes, and Mgaya et al. [29], where two out of twenty (2/20) isolates had *bla*_CTX-M_ with no detected genes of *bla*_TEM_ and *bla*_SHV_. A similar high prevalence of *bla*_TEM_ was observed in chicken droppings in Nairobi (46%), though significantly higher compared with this study [42].

## 5. Conclusion and Recommendation

This study found out that *Escherichia coli* isolated from selected broilers and culled layers of healthy chickens in Arusha and Mwanza regions were resistant to at least one antimicrobial agent used in this study. The study recommends continued antimicrobial surveillance in broiler and layer chickens and at large to the poultry farms and markets including those reared in individual households. Further research can be conducted to investigate whether the same resistance genes found in *E. coli* isolates in this study are similar to those that can be probably isolated from rectum swabs from the poultry farmers and their immediate environment.

## Figures and Tables

**Table 1:** Overall prevalence of ESBL-producing *Escherichia coli* obtained from Mwanza and Arusha regions in Tanzania.

Antimicrobial gene	Broiler	Layer	Total
*bla*_TEM_	13 (61.9)	8 (38.1)	100
*bla*_CTX-M_	0 (0.0)	2 (9.5)	9.5
*bla*_SHV_	0 (0.0)	0 (0.0)	0

**Table 2:** Overall antimicrobial resistance prevalence of the randomly selected *E. coli* isolates to the tested antimicrobial agents in Arusha and Mwanza (*n* = 204).

Sn	Antimicrobial agent	Frequency	%
1	Ertapenem	62	30.4
2	Chloramphenicol	110	53.9
3	Ceftriaxone	95	46.6
4	Ciprofloxacin	140	68.6
5	Ampicillin	204	100
6	Trimethoprim/sulfamethoxazole	182	89.2
7	Gentamicin	21	10.3

**Table 3:** Prevalence of resistance to selected antimicrobial agents among *Escherichia coli* isolates obtained from broiler and layer chickens in Arusha and Mwanza by chicken type.

Sn	Antimicrobial agent	Proportion of resistance *n* (%)		*p* value
		Broiler (*n* = 103)	Layer (*n* = 101)	
1	Ertapenem	32 (15.7)	30 (14.7)	0.832
2	Chloramphenicol	58 (28.4)	52 (25.5)	0.489
3	Ceftriaxone	53 (26.0)	21 (20.6)	0.158
4	Ciprofloxacin	71 (34.8)	69 (33.8)	0.925
5	Ampicillin	103 (50.5)	101 (49.5)	—
6	Trimethoprim/sulfamethoxazole	96 (47.1)	86 (42.2)	0.064
7	Gentamicin	13 (6.4)	8 (3.9)	0.269

Key: Sn = serial number.

**Table 4:** Overall antimicrobial resistance to different classes of antimicrobial agents of the randomly selected *E. coli* isolates obtained from broiler and layer chickens in Arusha and Mwanza.

Number of antimicrobial agent classes	Frequency	Percentage (%)
1	11	5.4
2	16	7.8
3	50	24.5
4	55	27
5	37	18.1
6	29	14.2
7	6	2.9

**Table 5:** Prevalence of multidrug resistance among 204 randomly selected *E. coli* isolated from broiler and layer chickens from Arusha and Mwanza.

MDR *E. coli* isolates	Number of classes of antimicrobial agents, *n* (%)				
Classes	3	4	5	6	7
*n* = 177	50 (28.2)	55 (31.1)	37 (20.9)	29 (16.4)	6 (3.4)

**Table 6:** Overall multidrug resistance patterns of *E. coli* isolates obtained from broiler and layer chickens from Arusha and Mwanza.

Antimicrobial combination	Number of isolates	Percentage	Number of antimicrobial classes
C, CIP, AMP	2	1.13	3
C, AMP, STX	16	9.04	3
CIP, AMP, CN	1	0.56	3
ETP, AMP, STX	1	0.56	3
CIP, AMP, STX	20	11.3	3
CRO, CIP, AMP	1	0.56	3
CRO, AMP, STX	9	5.08	3
C, CIP, AMP, STX	24	13.56	4
ETP, C, AMP, STX	1	0.56	4
C, CRO, AMP, STX	5	2.82	4
CIP, AMP, STX, CN	1	0.56	4
ETP, CIP, AMP, STX	3	1.69	4
CRO, CIP, AMP, STX	14	7.91	4
ETP, CRO, CIP, AMP	2	1.13	4
ETP, CRO, AMP, STX	5	2.82	4
C, CIP, AMP, STX, CN	5	2.82	5
ETP, C, CIP, AMP, STX	8	4.52	5
ETP, C, CRO, CIP, AMP	1	0.56	5
C, CRO, AMP, STX, CN	1	0.56	5
C, CRO, CIP, AMP, STX	13	7.34	5
CRO, CIP, AMP, STX, CN	2	1.13	5
ETP, CRO, AMP, STX, CN	1	0.56	5
ETP, CRO, CIP, AMP, STX	6	3.39	5
ETP, C, CRO, CIP, AMP, STX	28	15.82	6
ETP, CRO, CIP, AMP, STX, CN	1	0.56	6
ETP, C, CRO, CIP, AMP, STX, CN	6	3.39	7

**Table 7:** Resistance pattern of the ESBL-positive *E. coli*.

Antimicrobial agents combination	Number of isolates	%	Number of antimicrobial classes
AMP, STX, CN	1	4.76	3
CIP, AMP, STX	2	9.52	3
CRO, AMP, STX	5	23.81	3
C, CRO, AMP, STX	2	9.52	4
ETP, CRO,AMP, STX	1	4.76	4
C, CIP, AMP, STX, CN	1	4.76	5
C, CRO, CIP, AMP,STX	4	19.05	5
ETP, C, CIP, AMP, STX	1	4.76	5
CRO, CIP, AMP, STX, CN	1	4.76	5
ETP, CRO, CIP, AMP, STX	2	9.52	5
ETP, C, CRO, CIP, AMP, STX	1	4.76	6

## Data Availability

The data are available from the corresponding author upon request.
